# Author Correction: The U‑shaped association of serum iron level with disease severity in adult hospitalized patients with COVID‑19

**DOI:** 10.1038/s41598-021-96408-2

**Published:** 2021-08-16

**Authors:** Kentaro Tojo, Yoh Sugawara, Yasufumi Oi, Fumihiro Ogawa, Takuma Higurashi, Yukihiro Yoshimura, Nobuyuki Miyata, Hajime Hayami, Yoshikazu Yamaguchi, Yoko Ishikawa, Ichiro Takeuchi, Natsuo Tachikawa, Takahisa Goto

**Affiliations:** 1 Department of Anesthesiology and Critical Care Medicine, Yokohama City University School of Medicine, 3‑9, Fukuura, Kanazawa‑ku, Yokohama, 236‑0004 Japan; 2 Department of Anesthesiology, Yokohama Municipal Citizen’s Hospital, Yokohama, 221‑0085 Japan; 3 Department of Emergency Medicine, Yokohama City University School of Medicine, Yokohama, 236‑0004 Japan; 4 Department of Gastroenterology and Hepatology, Yokohama City University School of Medicine, Yokohama, 236‑0004 Japan; 5 Department of Infection Medicine, Yokohama Municipal Citizen’s Hospital, Yokohama, 221‑0085 Japan

Correction to: *Scientific Reports* 10.1038/s41598-021-92921-6, published online 28 June 2021

In the original version of this Article Natsuo Tachikawa was incorrectly affiliated with ‘Department of Gastroenterology and Hepatology, Yokohama City University School of Medicine, Yokohama, 236-0004, Japan’. The correct affiliation is listed below.

Department of Infection Medicine, Yokohama Municipal Citizen’s Hospital, Yokohama, 221-0085, Japan

The original Article has been corrected.

