# Supplementary material for: The U-shaped association of serum iron level with disease severity in adult hospitalized patients with COVID-19
Source: Sci Rep. 2021 Jun 28;11:13431. doi: 10.1038/s41598-021-92921-6 (PMC8238936; doi:10.1038/s41598-021-92921-6)

**Supplementary Figure. 1**

Comparisons of (A) serum iron, (B) transferrin saturation (TSAT), (C) total iron binding capacity (TIBC), and (D) ferritin levels between male and female patients.


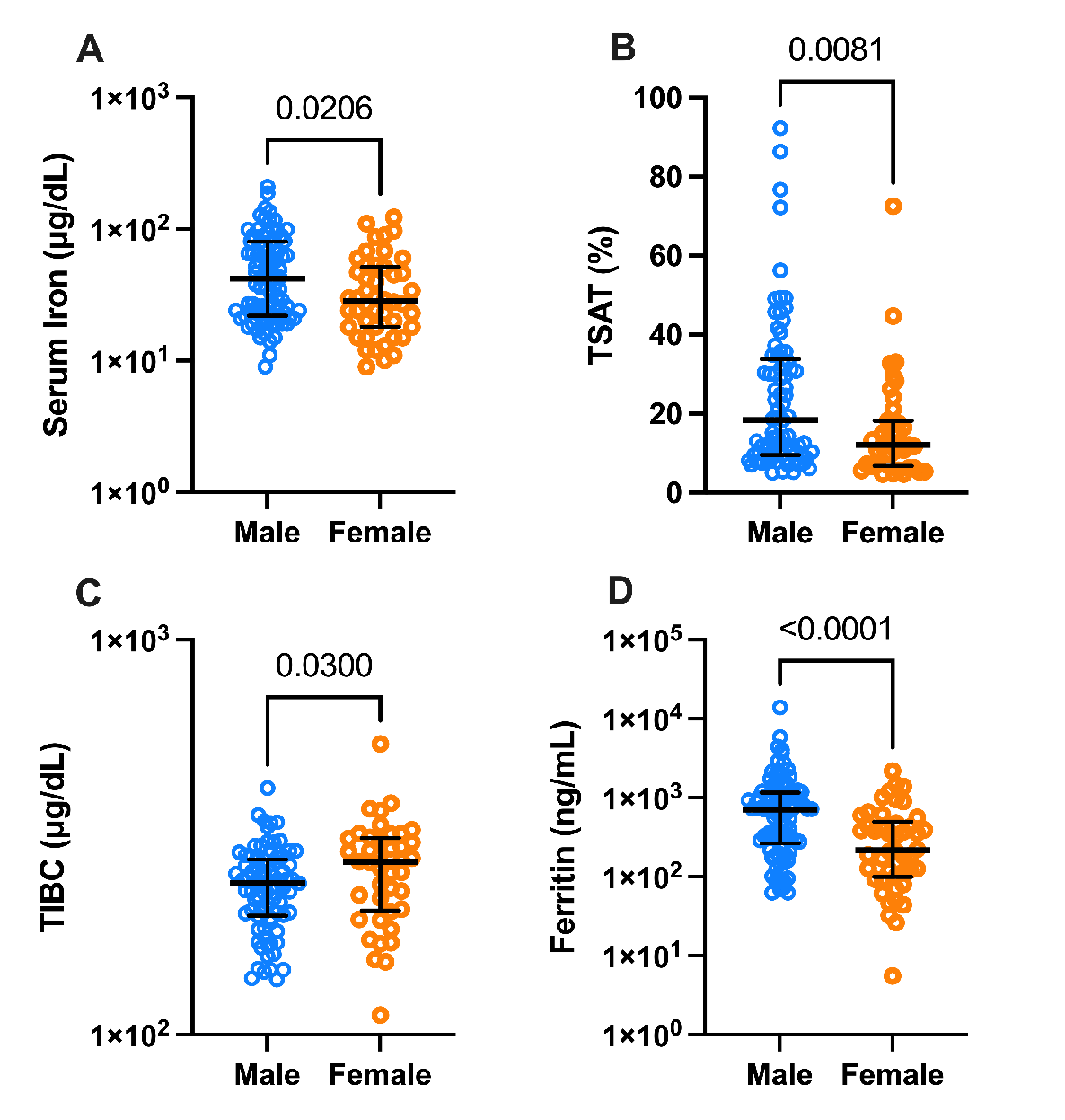


(E) Serum iron and (F) TSAT levels in male patients.


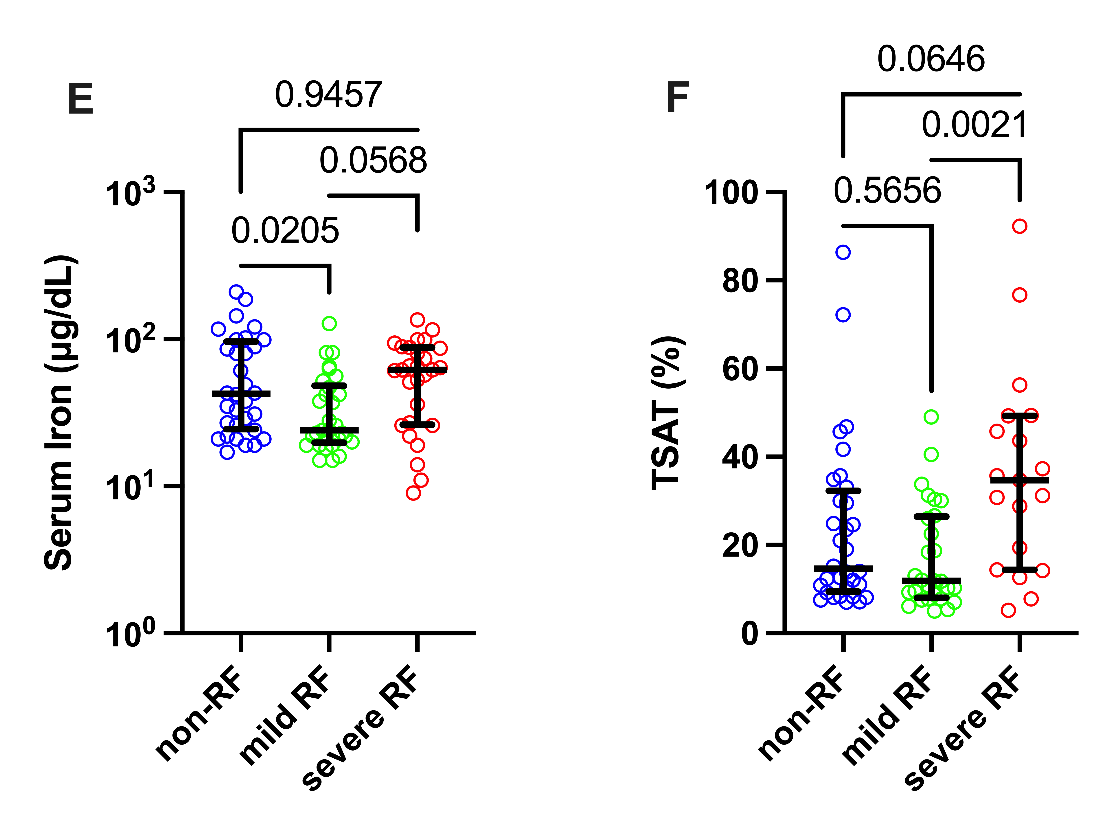

(G) Serum iron and (H) TSAT levels in female patients.


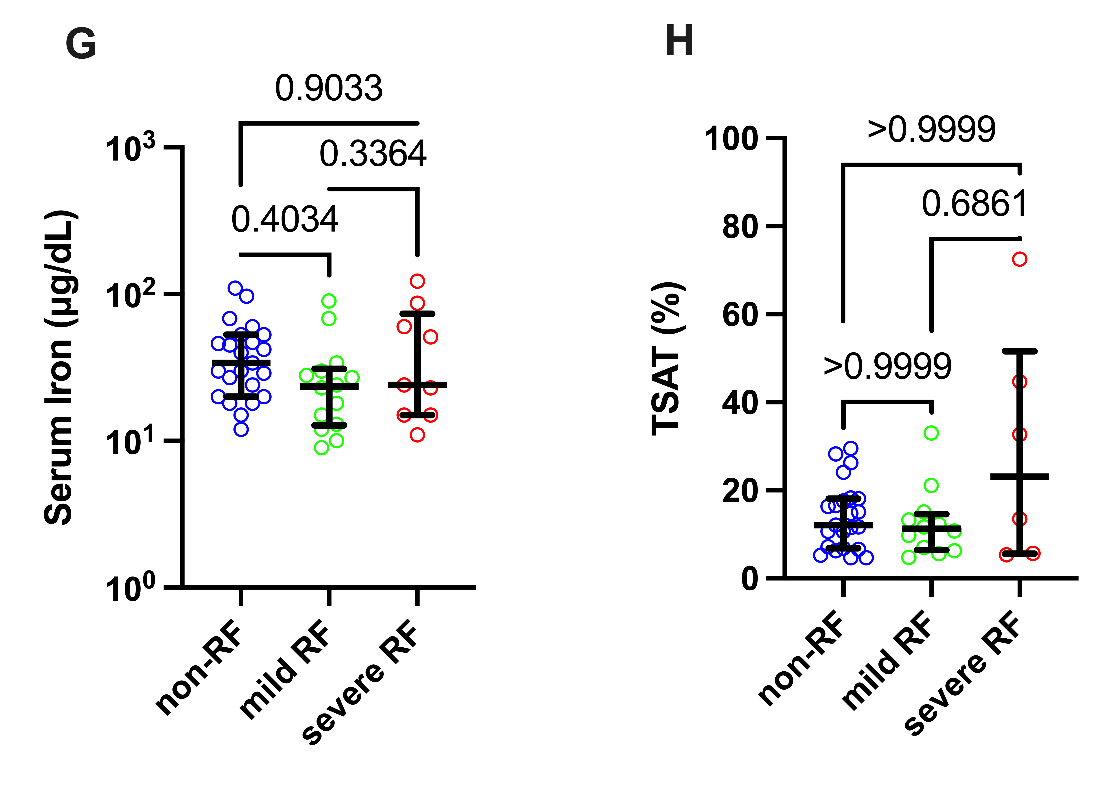

Supplement: Supplementary file 1 — Supplementary Figure 1. [file 41598_2021_92921_MOESM1_ESM.docx]
