# Supplementary material for: The U-shaped association of serum iron level with disease severity in adult hospitalized patients with COVID-19
Source: Sci Rep. 2021 Jun 28;11:13431. doi: 10.1038/s41598-021-92921-6 (PMC8238936; doi:10.1038/s41598-021-92921-6)

**Supplementary Figure. 2**

Comparisons of serum iron levels between survived and non-survived patients.


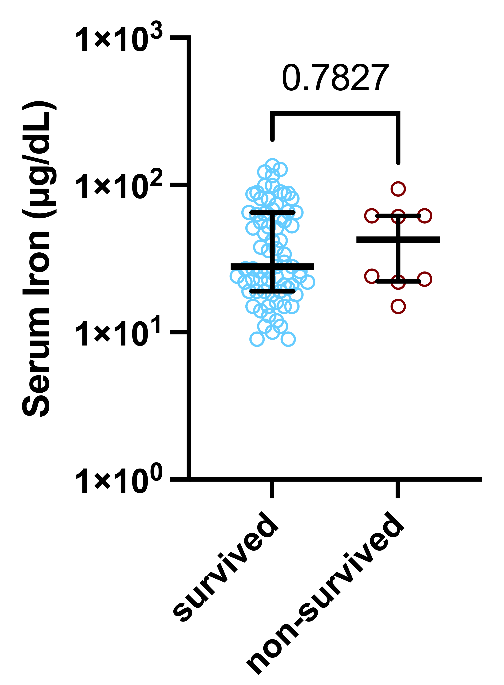

Supplement: Supplementary file 2 — Supplementary Figure 2. [file 41598_2021_92921_MOESM2_ESM.docx]
